# A wearable multi-modal acoustic system for breathing analysisa)

**DOI:** 10.1121/10.0009487

**Published:** 2022-02-15

**Authors:** Lloyd E. Emokpae, Roland N. Emokpae, Ese Bowry, Jaeed Bin Saif, Muntasir Mahmud, Wassila Lalouani, Mohamed Younis, Robert L. Joyner

**Affiliations:** 1 LASARRUS Clinic and Research Center, Baltimore, Maryland 21220, USA; 2 Department of Computer Science and Electrical Engineering, University of Maryland, Baltimore County, Baltimore, Maryland 21250, USA; 3 Richard A. Henson Research Institute, TidalHealth Peninsula Regional, Salisbury, Maryland 21801, USA

## Abstract

Chronic obstructive pulmonary disease (COPD) is the third leading cause of death worldwide with over 3 × 10^6^ deaths in 2019. Such an alarming figure becomes frightening when combined with the number of lost lives resulting from COVID-caused respiratory failure. Because COPD exacerbations identified early can commonly be treated at home, early symptom detections may enable a major reduction of COPD patient readmission and associated healthcare costs; this is particularly important during pandemics such as COVID-19 in which healthcare facilities are overwhelmed. The standard adjuncts used to assess lung function (e.g., spirometry, plethysmography, and CT scan) are expensive, time consuming, and cannot be used in remote patient monitoring of an acute exacerbation. In this paper, a wearable multi-modal system for breathing analysis is presented, which can be used in quantifying various airflow obstructions. The wearable multi-modal electroacoustic system employs a body area sensor network with each sensor-node having a multi-modal sensing capability, such as a digital stethoscope, electrocardiogram monitor, thermometer, and goniometer. The signal-to-noise ratio (SNR) of the resulting acoustic spectrum is used as a measure of breathing intensity. The results are shown from data collected from over 35 healthy subjects and 3 COPD subjects, demonstrating a positive correlation of SNR values to the health-scale score.

## I. INTRODUCTION

The COVID-19 pandemic has elevated attention to serious respiratory diseases not only because of the acute symptoms but also due to the stress it imposes on the healthcare system. Comorbidities are associated with the worst outcomes for the COVID-19 disease. Chronic obstructive pulmonary disease (COPD) is a leading cause of morbidity and mortality worldwide and is associated with substantial and increasing economic and social burdens.^1^ Patients with preexisting COPD who are diagnosed with COVID-19 have a more than three times higher risk of severe symptoms and mortality.^2^ The prevention and early recognition of COPD exacerbation is important for reducing severe symptoms through early intervention. The clinical course of COPD is marked by escalating symptoms, which lead to an increased rate of hospitalizations.^3,4^ The main symptoms of COPD include dyspnea (i.e., shortness of breath), coughing, and the production of sputum. The recent data show that the readmission rate for COPD patients approaches 23%,^5^ which creates a huge burden on the healthcare system. Given that most exacerbations can be treated at home with the proper medication, the early detection of COPD symptom exacerbations enables major reductions in COPD patient readmissions,^6^ which also limits the need for frequent healthcare provider visits and, therefore, avoids the unnecessary risk of exposure to COVID-19. This strategy aligns with the goal of protecting COPD patients from being exposed to COVID-19, which is a priority for pulmonary-care providers. The standard adjuncts used to assess lung function (e.g., spirometry, plethysmography, and CT scan) require oversight by medical experts, are time consuming, and cannot be used in remote patient monitoring of an acute exacerbation. Currently, there are no tools that enable the remote monitoring of the lung function of COPD patients and detect acute symptoms, which can indicate an increased severity in their symptoms (e.g., resulting from COVID-19) to prevent hospitalization. Also, it is common for future predictions of COPD exacerbations to rely on the history of exacerbations from previous years with questionnaires that aim to quantify changes in the symptoms between doctor visits.^7^ However, the response to the questionnaires is not reliable as it incurs a level of subjectivity and does not capture the symptom exacerbation in a timely manner. Moreover, the current systems, which aim to examine lung function, rely mainly on a single modality such as using data from a phonocardiogram (PCG) or digital stethoscope, measured at a single point.^8,9^

The aforementioned issues have motivated us to extend our Intel (Santa Clara, CA) award-winning and patent-pending wireless wearable multi-modal electroacoustic (WearME) system to capture patient exacerbation changes on both lungs in real time and detect breathing anomalies. WearME employs a body area sensor network with each sensor-node having a multi-modal sensing capability, such as a digital stethoscope, electrocardiogram (EKG) monitor, thermometer, and goniometer. Leveraging the therapeutic potential of our acoustic sensing capability could provide a physiological link for the provider to COPD patients quarantining at home to prevent exposure to COVID-19. This will enable medical intervention that could limit the severity of a COPD exacerbation. In this paper, we present the breathing analysis results from a pilot study of 35 healthy subjects and 3 COPD subjects captured with our WearME system. We define a signal processing metric for analyzing the acoustic breathing features, which can be used to infer certain levels of COPD severity. This research contribution can be used for further analysis against the gold standard of pulmonary function tests (PFTs). In essence, our WearME system can be extended to capture the indicators of physiologic baseline and acute changes during exacerbation in patients with known COPD severity as per the American Thoracic Society (ATS)/European Respiratory Society (ERS) guidelines^10^ through correlation analysis to spirometric PFTs and COPD approved questionnaires.^11^ The paper is organized as follows. Section II discusses related work in the literature and summarizes our WearME system and the modalities used in our analysis. Section III presents the results of our pilot study, and Sec. IV concludes the paper and discusses future work.

## II. SYSTEM DESIGN

The WearME system uses a body area sensor network with each node having a suite of sensors (digital stethoscope for auscultation, ECG monitor, temperature sensor, and body posture tracker). The WearME system can be used either with the shirt option or strap option. The shirt is designed with an array of internal pockets, which are used for easy sensor placement. Careful consideration has been taken in our prototype implementation to ensure that the design does not impede the patient's movement ability and range of motion. The WearME sensors are also designed to be reusable, rechargeable, and should be removed from the shirts prior to washing or dry cleaning. The WearME sensor modalities have been validated against other solutions in the market, e.g., Eko Health (Duo ECG and Stethoscope, Oakland, CA), and WAND Temperature sensor (American Fork, UT). More information can be found in our previous publications.^12–14^ Furthermore, we designed our user interface (UI) software to facilitate the operation by users within the COPD demographics and provide built-in instructions to ensure reliable data measurements.

**FIG. 1.**
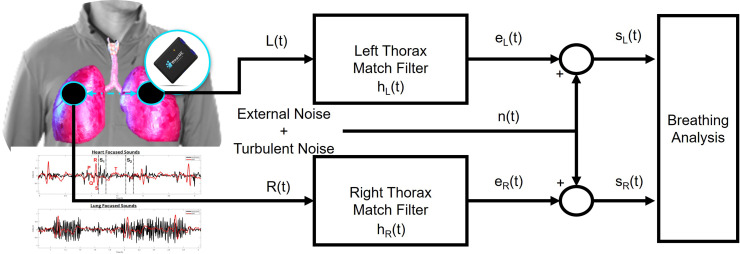
(Color online) An illustration of our WearME system, used for bilateral lung data processing. WearME employs a body area sensor network with each sensor-node having a multi-modal sensing capability, such as a digital stethoscope, ECG, thermometer, and goniometer. In this paper, we mainly focus on our acoustic sensing and lung function metric, namely, the signal-to-noise ratio (SNR) in which the noise also reflects the turbulence caused by airflow obstruction.

Figure 1 illustrates our approach for using the WearME system to detect COPD symptoms by assessing the breathing regularity. The basic idea is to use the acoustic sensors to capture the lung sound and analyze it for anomalies. We can define our bilateral acoustic system as a function of two received signal models $s_{l}\left(t\right)$ and $s_{r}\left(t\right)$ as

$$s_{l}\left(t\right)=l\left(t\right)*h_{l}\left(t\right)+n\left(t\right)=\sum_{j=1}^{J}A_{j}l\left(t-\tau_{j}\right)+n\left(t\right),$$ (1)

$$s_{r}\left(t\right)=r\left(t\right)*h_{r}\left(t\right)+n\left(t\right)=\sum_{k=1}^{K}A_{k}r\left(t-\tau_{k}\right)+n\left(t\right),$$ (2)

where “∗” is the convolution operator, $l\left(t\right)$ and $r\left(t\right)$ represent the original left and right thorax acoustic data, respectively, $h_{l}\left(t\right)$ and $h_{r}\left(t\right)$ are the match filters for the left and right lungs, respectively, $n\left(t\right)$ is the additive uncorrelated noise signal, $\left(\tau_{j},A_{j}\right)\;\text{and}\;\left(\tau_{k},A_{k}\right)$ correspond to the time delays and attenuation factors for the impulse responses of the respective match filters with the *j*th and *k*th filter taps.

### A. Breathing metric: Signal-to-noise ratio

We adopt the signal-to-noise ratio (SNR) as a metric for measuring the breathing intensity of the subject. The SNR is computed by taking the ratio of the signal power to the noise power and is a measure of the breathing intensity. This is described mathematically as

$${\text{SNR}}_{\text{dB}}=10\;\;{\text{log}}_{10}\left(\frac{P_{S}}{P_{N}}\right)=P_{S,\text{dB}}-P_{N,\text{dB}},$$ (3)

where $P_{S}$ is the signal power in watts, $P_{N}$ is the noise power in watts, $P_{S,\mathrm{dB}}$ is the signal power in decibels, and $P_{N,\mathrm{dB}}$ is the noise power in decibels. The signal power will be taken from each of the respective acoustic signals $s_{l}\left(t\right)$ and $s_{r}\left(t\right)$ and defined to be the normal sound power during breathing activity. The noise power is the sum of the ambient noise (background sound) and acoustic noise caused by airflow obstruction. All COPD patients have varying levels of airflow obstruction, which induces turbulence.^15^ Such turbulence is captured as noise in our acoustic measurement. Hence, an increase in airflow obstruction will decrease the SNR.

**FIG. 2.**
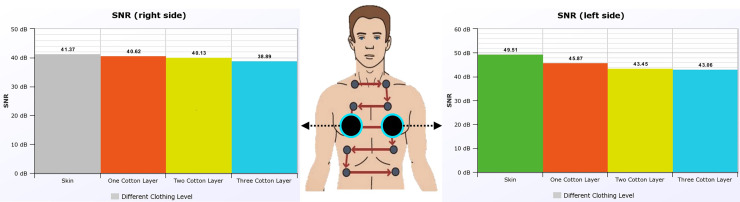
(Color online) The bilateral SNR with varying material thickness. For this subject, the left thorax breathing level has an increase of approximately 8 dB in the SNR over the right thorax.

Figure 2 shows the output of the SNR on a healthy subject with and without skin contact. The results show a decrease in the SNR as we increase the fabric thickness, which is expected. The SNR is also higher on the anterior left thorax over the anterior right thorax because of the presence of heart sounds.

## III. PILOT STUDY

**FIG. 3.**
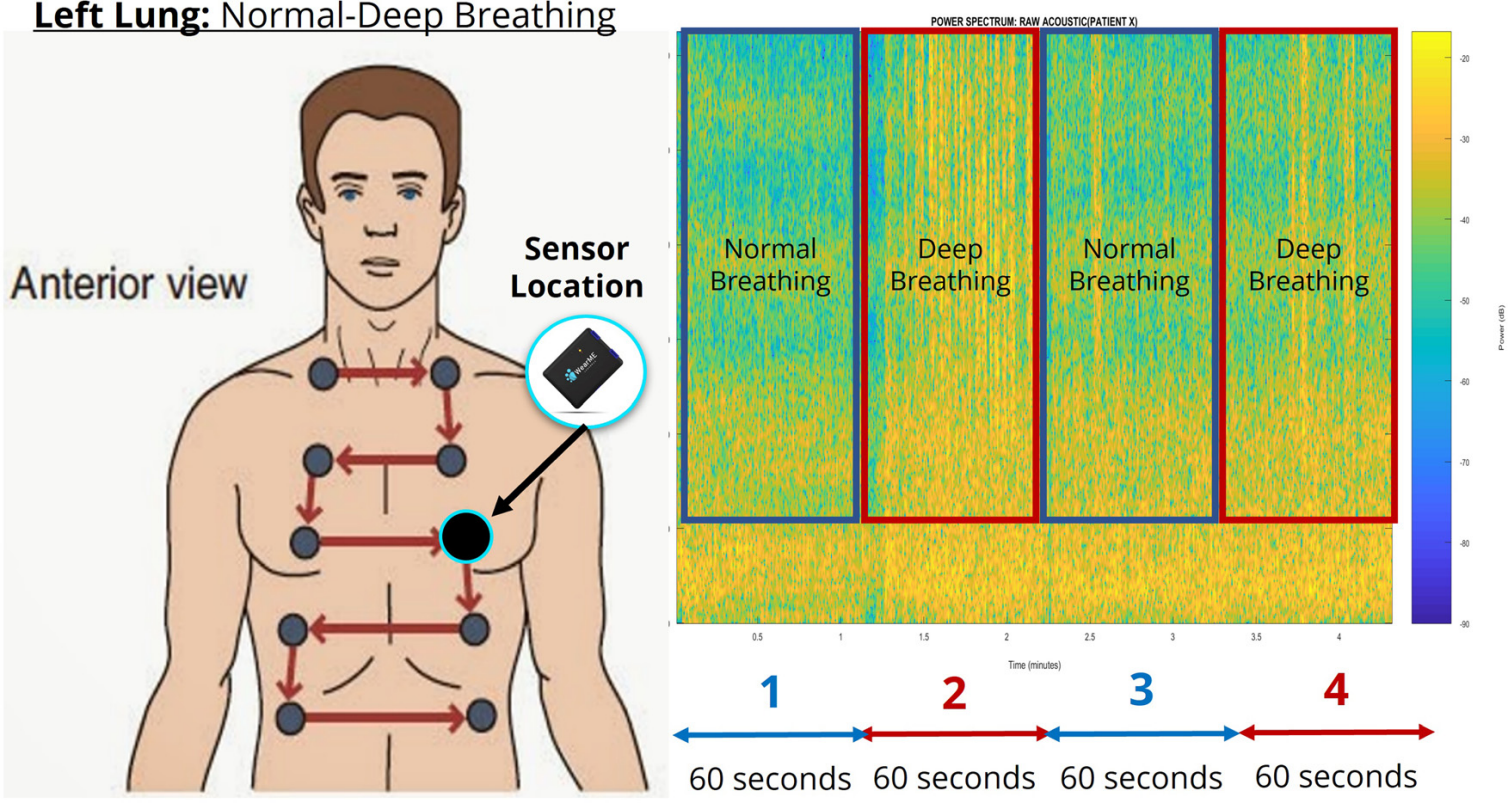
(Color online) The unilateral breathing sequence performed during the pilot testing of a 72-year-old male healthy subject. The power spectrum analysis shows the distinguishable frequency features between normal and deep breathing exercises.

**FIG. 4.**
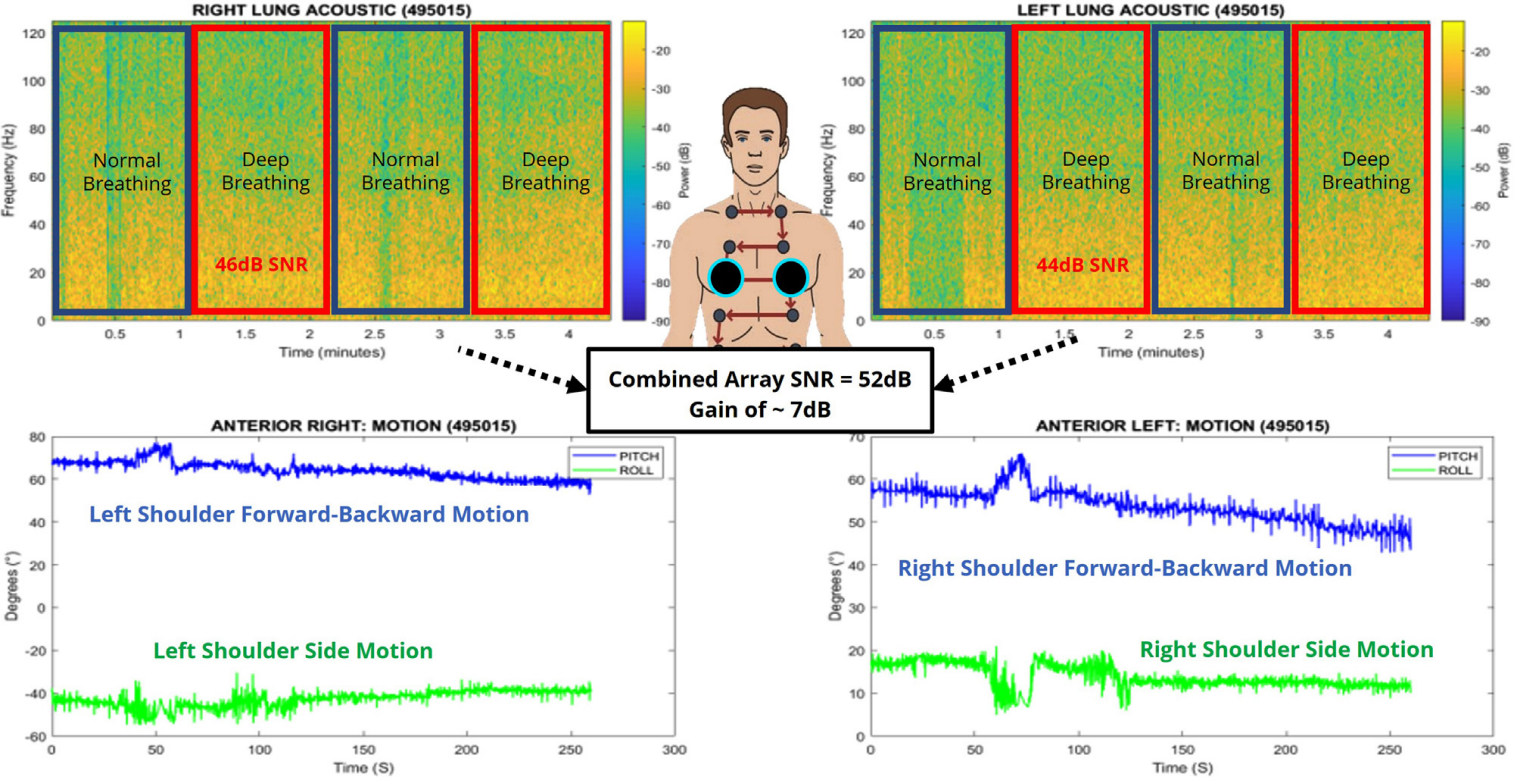
(Color online) The bilateral breathing power spectrum of both anterior lungs simultaneously from a 23-year-old healthy subject. The motion was also captured and depicts slight forward-backward motions during the breathing assessment. The subject was seated and at rest during the assessment. The combined array processing yields a gain in the SNR of ∼7 dB.

A pilot study was conducted in the summer of 2021 with 35 healthy baseline subjects and 3 COPD subjects. We attended running events in the District of Columbia, Maryland, and Virginia (DMV) areas to obtain baseline data from healthy subjects. Each participating subject performed a series of breathing exercises for approximately 4 min, which consisted of normal and deep breathing transitions as shown in Fig. 3. For unilateral data collection, we placed our WearME sensor at the anterior left thorax for one normal-to-deep breathing cycle before switching the sensor to the anterior right thorax for the next normal-to-deep breathing cycle. Meanwhile, for bilateral data collection (Fig. 4), we used two WearME sensors, which were placed anteriorly on the left and right thorax to assess both lungs simultaneously, which did not require moving the sensors. We also captured the metadata in the form of questionnaires pertaining to the health of each participant; these data include answers to the questions pertaining to their fitness level and health, which we used to generate a health-scale score in the range of 1–10 (with “10” denoting excellent health). The metadata consisted of two tabs, one with questionnaires for subjects without any underlying respiratory problems and the second with questionnaires to collect the respiratory symptoms from subjects who have been diagnosed with COPD. Each subject was given a unique identification (ID) number. We also collected other information such as their age and gender.

**FIG. 5.**
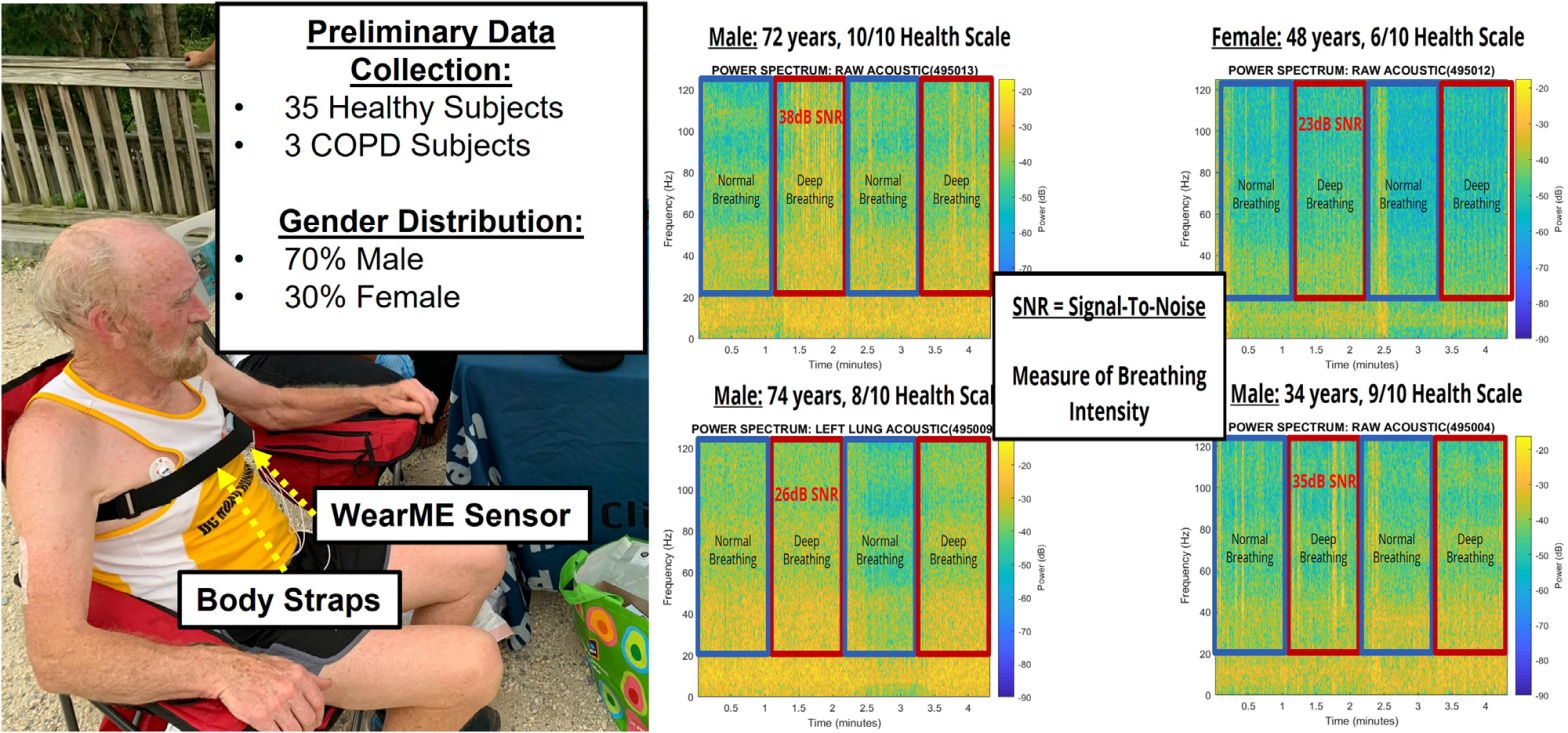
(Color online) An image of a healthy baseline runner using our WearME system for breathing analysis (left). The sample subject breathing distribution (right) shows a higher SNR for subjects with a higher health scale. We collected data from 35 healthy subjects and 3 COPD subjects with comparable results.

**FIG. 6.**
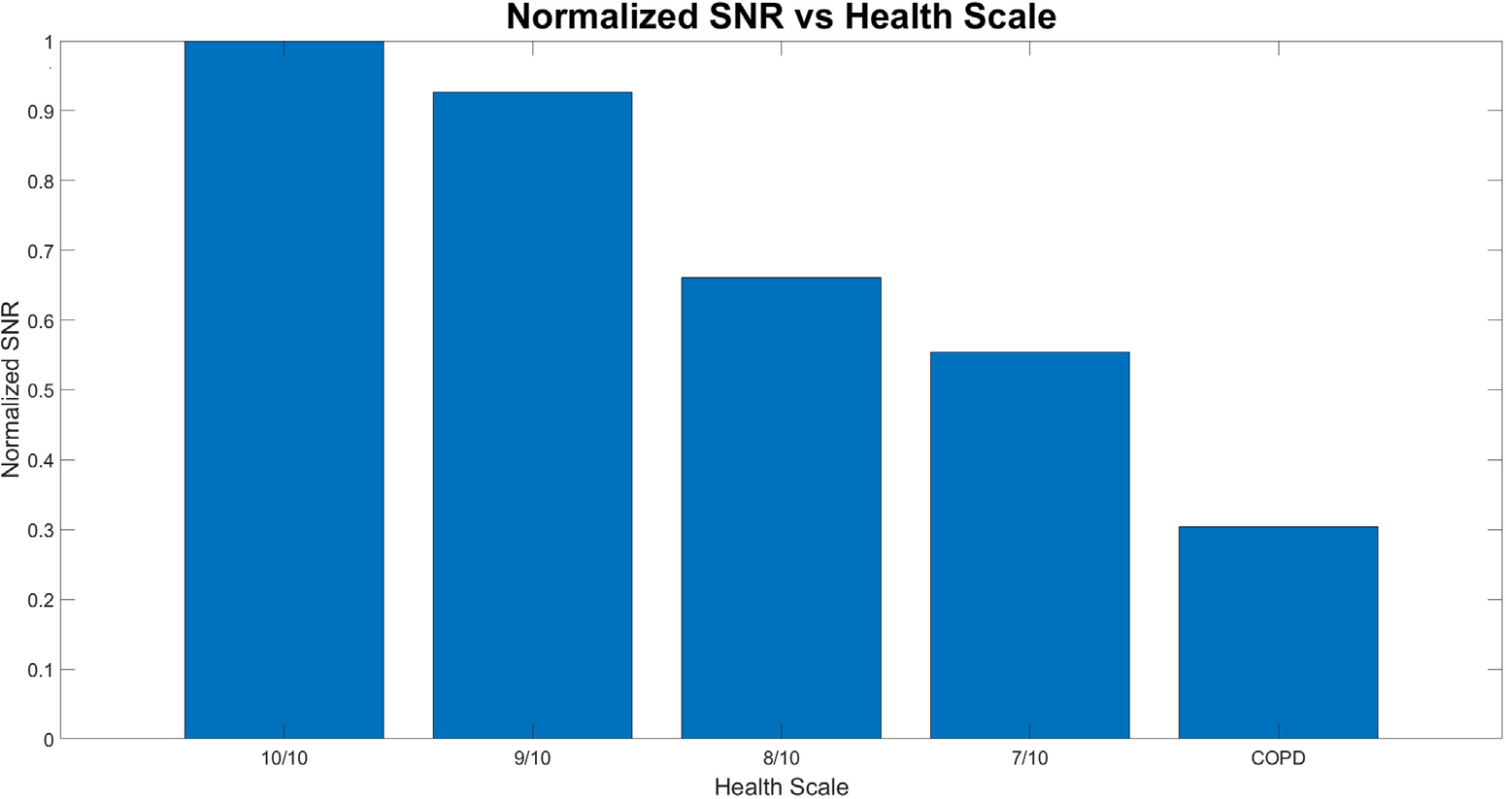
(Color online) The distribution of the normalized SNR values to the respective health scale averaged over the recorded data sets. The results show a positive correlation to the health scale to the normalized SNR of the subject. The average health scale for the three COPD subjects was found to be 5.5/10.

The results obtained show the unique frequency features on both lungs, which can be explored to better understand the contribution of each lung toward the subject's breathing capacity. This effect can be captured by determining the SNR of the captured acoustic signal. The SNR is computed by taking the ratio of the signal power to the noise power and is a measure of the breathing intensity. Furthermore, we note that through array processing, we were able to achieve the array gain on the order of 7 dB through bilateral processing over the unilateral case as shown in Fig. 4. We also captured the body motion during the assessment, which allows us to further filter out the noise associated with body movement to improve our acoustic processing. A sample distribution of our preliminary data is shown in Fig. 5, which depicts higher SNR values for subjects with higher health scales, whereby approximately 70% of the subjects are male and 30% are female. It is important to note that we only used the body strap option in this study. From our analysis, we saw a positive correlation to the normalized SNR values to the health-scale score obtained from the metadata questions, and this is illustrated in Fig. 6. The distribution of the SNR values was averaged amongst each health-scale group and normalized accordingly for comparison. Furthermore, the average health scales for the COPD subjects who participated in our study were found to be approximately 5.5 out of ten.

## IV. CONCLUSION

In this paper, we have presented a wearable multi-modal system for breathing analysis, which can be used in quantifying various airflow obstructions. Our WearME system employs a body area sensor network with each sensor-node having a multi-modal sensing capability, such as digital stethoscope, EKG monitor, thermometer, and goniometer. The SNR of the resulting acoustic spectrum is used as a measure of the breathing intensity. The results are shown from data collected from over 35 healthy subjects and 3 COPD subjects; the results demonstrate a positive correlation of the SNR values to the health-scale scores. A treatment strategy that includes our WearME device may allow providers to treat COPD patients at home. This could reduce the need for COPD patients to risk exposure to COVID-19 from an office or emergency room visit, thus, providing overall improved care for the COPD patient and reducing the economic burden of the disease on the patient and healthcare system. For future work, we plan to extend the scope of the data collection and analysis by recruiting COPD subjects with known severity and monitoring them over an extended period, e.g., 3–5 months, to study the WearME system acceptability and the performance of our breathing metric for follow-on clinical studies, and this includes studying the statistical distribution and correlation of each subjects SNR values to the gold standard PFTs.
